# Discovery of a bacteriophage sequence in a mite genome assembly reveals bacterial contamination and opens new possibilities for exploring arthropod symbionts

**DOI:** 10.1099/mgen.0.001520

**Published:** 2025-10-08

**Authors:** Elisa Luque-Jiménez, Antonio Moreno-Rodríguez, Andrés Garzón, Alejandro Rubio, Antonio J. Pérez-Pulido

**Affiliations:** 1 Andalusian Centre for Developmental Biology (CABD, UPO-CSIC-JA), Faculty of Experimental Sciences (Genetics Area), University Pablo de Olavide, 41013, Seville, Spain

**Keywords:** *Acinetobacter*, contamination, mite, parthenogenesis, *Rickettsia*, symbiont

## Abstract

While studying the integration site of a bacteriophage associated with the bacterium *Acinetobacter baumannii*, we found a genome assembly of the mite *Oppiella nova* that contained a homologous sequence of this locus. We initially thought of horizontal gene transfer, but it actually uncovered the contamination of 41 genome fragments with a total of 2.28 Mb. This has allowed us to assemble a new genome of the species *Acinetobacter guillouiae*, which could be a symbiont of the mite, based on the identification of genes potentially related to the diet of this arthropod. This contamination has been unknowingly spread, at least in another article in which authors studied a gene associated with antibiotic resistance. These results recommend the re-assembly of the *O. nova* genome and show how current sequencing databases have information to study microbial symbionts without the need for new experimentation.

**Impact Statement**

We have found significant bacterial contamination within the genome assembly of a mite of the species *Oppiella nova*, which had begun to spread in the scientific literature. Specifically, the mite’s genome assembly contained the complete genome of a new strain of the bacterium *Acinetobacter guillouiae*, which could help the host feed, and part of the genome of a *Rickettsia*, which could be involved in the mite’s asexual reproduction. This discovery, made while investigating the origin of a bacteriophage, has led to the proposal of new bacterial–mite symbiotic relationships and opens up a new avenue for studying these relationships using genomes stored and available in public databases.

## Data Summary

The identifiers for all data analysed are available in the manuscript and in the SI Appendix. Details on the methodology used, including written command lines, are available in the Supplementary Material. The new assemblies and results from Kraken and MUMmer are available in the following repository: https://doi.org/10.5281/zenodo.15242142, as well as at BioProject: PRJNA1328477.

## Introduction

When the first tardigrade genome, of the species *Hypsibius dujardini*, was sequenced in 2015, it was shown that one-sixth of its genes had been acquired through horizontal gene transfer, mainly of bacterial origin [1]. Subsequent studies were quickly published, demonstrating that the origin was none other than contamination [2,3]. This effectively corrected the initial interpretation and prevented the error from spreading.

Contamination of public sequence databases is a relatively common problem that is sometimes not adequately identified [4,5]. It can arise from various sources, including sample handling, sequencing reagents or co-extracted organisms. This is especially common in samples of small organisms or environmental samples, where it is difficult to isolate the target organism [6]. It is therefore essential to filter this data and distinguish between actual cases of horizontal gene transfer and contamination and to characterize the nature of the latter when present, to better understand the organisms under study.

While studying the sequence of a bacteriophage integrated into the genome of a strain of the bacterium *Acinetobacter baumannii* [7], we discovered the same integration region in the genome assembly of the mite *Oppiella nova*, published in 2021 [8]. It is an oribatid mite commonly found on decaying organic matter that mainly feeds on fungal hyphae, thus playing an important role in the trophic relationships that occur during leaf litter decomposition [9]. This species reproduces asexually, so the authors studied the conservation of its genes in relation to another mite that is evolutionarily close but reproduces sexually, *Medioppia subpectinata* (homotypic synonym: *Oppiella subpectinata*).

This observation prompted us to investigate whether the presence of this sequence was the result of horizontal gene transfer or contamination and finally prove that it was the latter. However, the discovery has allowed us to study bacteria that may be symbiotic with the mite *O. nova*. One of them, *Acinetobacter guillouiae*, could help the mite with its feeding. The other, within the *Rickettsiales* order, could be interfering with the mite’s reproduction.

## Methods

### Initial similarity search

The *A. baumannii* genes for *ssrA* and a tyrosine-type recombinase/integrase (GenBank: NZ_CAJHGJ010000003.1; locus_tag: JO898_RS04190 and JO898_RS04195) were compared against the default non-redundant database in the NCBI blastn web application, excluding *Acinetobacter* (taxid:469).

The initially found contaminated contig (GenBank: OC914832.1; CAJPVJ010000007.1) was annotated with the prokaryote-specific gene finder, Bakta v1.10.3 with the full database [10]. Finally, the prophage was predicted using PHASTEST Upgrade 4 [11].

### Assessment of contamination

Two independent protocols were used to analyse the level of contamination by *A. guillouiae*. Initially, the sequences of the *O. nova* assembly were compared with the 42 available *A. guillouiae* assemblies to identify contaminated contigs. A contig of a minimal length of 3.5 kb was considered to belong to *A. guillouiae* when 90% of its sequence showed at least 95% identity to a sequence of the bacterium. The presence of this contamination was confirmed through a comparative analysis of *O. nova* contigs with those of *A. guillouiae* strain 13-3 (GenBank: CP142740.1) using the software MUMmer v4.0.1 [12].

Then, the raw data were subjected to analysis to ascertain the origin of the contamination. To this end, the same parameters used by the authors of the original manuscript were used to filter the reads, using Trimmomatic v0.39 [13], Fastp v1.0.1 [14] and NxTrim v0.4.3 [15]. After the processing of raw data, the Kraken v2.1.2 algorithm was used to identify the bacterial taxa that were responsible for the contamination present in the now processed data [16]. For this purpose, a specialized database containing all bacterial RefSeq sequences from the NCBI Genome database was previously constructed, as well as archaea, plants and fungi.

### Genome assembly

Reads were mapped against the *O. nova* genome, excluding contigs contaminated by *A. guillouiae*, using HISAT2 v2.2.1 [17]. Subsequently, the unmapped reads were subjected to positive selection using SAMtools v1.21 [18]. This process led to the elimination of all reads that belonged to *O. nova*. Then, a *de novo* assembly was performed using the Unicycler v0.5.1 algorithm [19], with default parameters, to ensure optimal performance.

After the acquisition of the draft genome, gene prediction was made using Bakta. Due to the possibility of genome mixing from different bacterial species, these predicted genes were functionally annotated with eggNOG-mapper v2.1.12 [20]. The functional annotation was then used for taxa assignment, and three groups were created (*Moraxellaceae*, *Rickettsiales* and others) using a PERL script based on the closest seed hit from the eggNOG-mapper annotation.

Finally, to obtain the complete genome of *A. guillouiae*, *Moraxellaceae* family-specific contigs were used in comparison with closed genomes of the species. The assembly was executed using the RagTag v2.1.0 tool [21]. BUSCO v6.0.0 was used to determine the degree of completeness of the assembled genomes [22]. The databases acinetobacter_odb12 and rickettsiales_odb12 were used as references. A new gene prediction and functional annotation of the genes in the final genome were carried out, in accordance with the previously established methodology.

### Molecular phylogeny

To obtain the phylogeny of *A. guillouiae*, a bacterial pangenome of this species was built for the purpose of selecting the core genes. To achieve this objective, the genomes were annotated with Bakta, and a core gene alignment was generated by Panaroo v1.5.2 [23], with MAFFT v.7.526 as the designated aligner [24]. Then, phylogeny was built using the IQ-TREE v2.4.0 algorithm with bootstrap 1000 [25], based on the aligned core genome.

In order to build the evolutionary history of the order *Rickettsiales*, the 23S ribosomal RNA sequences contained in the silva v.138.2_LSURef_NR99 database for this taxon were initially selected [26]. A comparison of all 23S rRNA sequences against contigs classified as *Rickettsiales* from *O. nova* was conducted using blastn v.2.16.0. The best hit was identified as the sequence of 23S rRNA of this *Rickettsia*. A multiple alignment was subsequently generated using MAFFT with this sequence and the remaining 23S rRNA sequences from the taxon *Rickettsiales*. The phylogenetic tree was constructed from this alignment by IQ-TREE, using a bootstrap of 1000 and the model GTR+F+I+G4.

The R packages ggtree v3.12.0 and ggimage v0.3.3 were used to visualize the molecular phylogenies and the corresponding metadata of the strains.

## Results

### A bacteriophage in a mite’s genome

*Acinetobacter* is a genus of opportunistic pathogenic bacteria isolated from soil, water and the microbiota of humans, other animals and plants [27]. We have recently described a warfare between two phages in *A. baumannii* [7], and while analyzing the sequence of the integrase of one of these phages, we found a contig from the genome assembly of the mite *O. nova* (BioSample: SAMEA7198734). This contig contained a region with 93% identity to the tmRNA gene (*ssrA*) used by the phage integrase to integrate into the chromosome of *A. baumannii*.

**Fig. 1.**
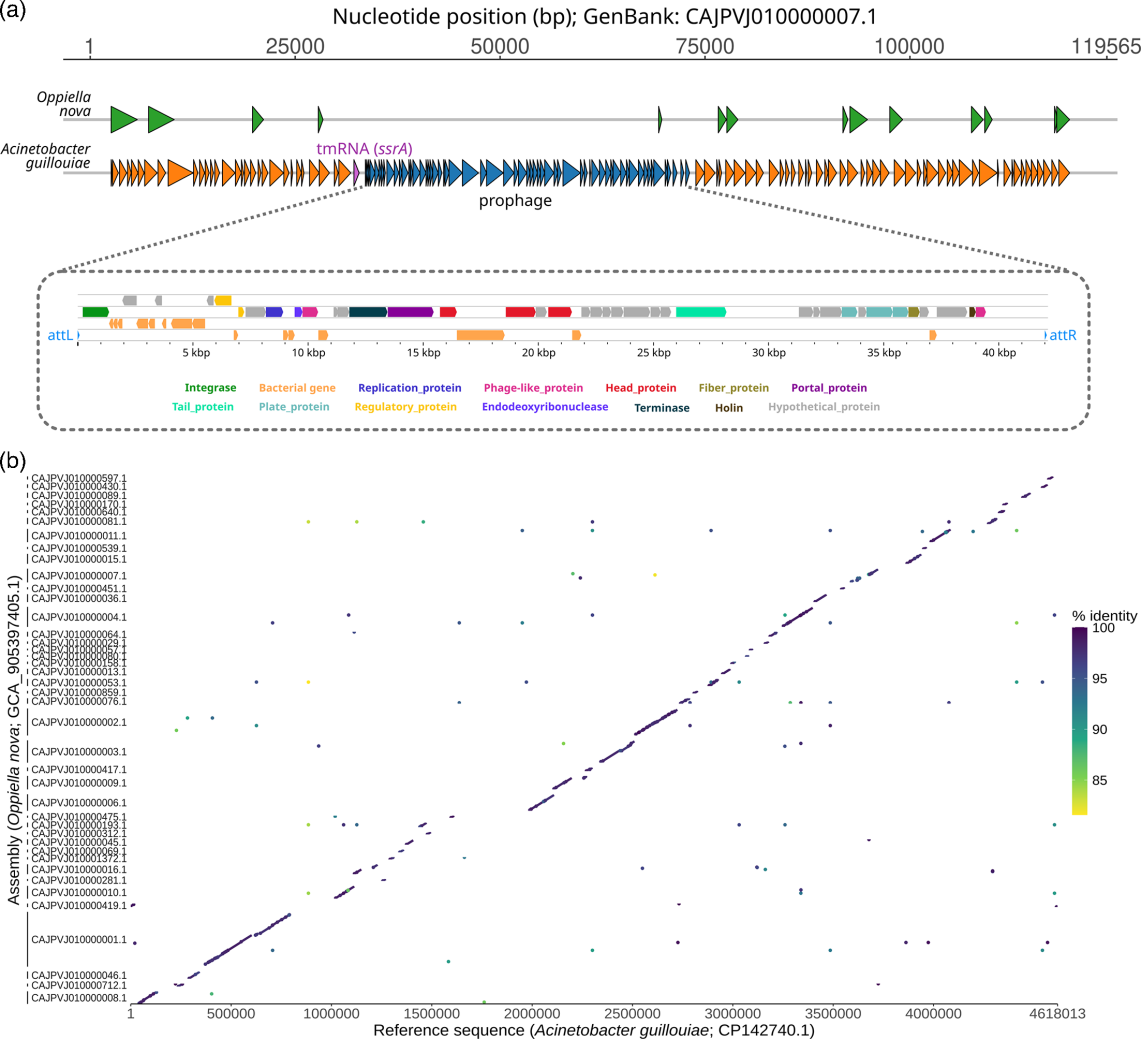
*Acinetobacter* contamination detected in *O. nova* contigs. (**a**) Contaminated contig of *O. nova* initially found. Genes predicted in the mite genome annotation are shown above (green). Prokaryotic genes predicted in the same contig are shown below (orange), including genes belonging to a prophage (blue), whose detailed annotation is shown in the zoom view. (**b**) Dotplot showing the similarity between the contaminated *O. nova* contigs and a genome of *A. guillouiae*.

Our first hypothesis was to explain this result by horizontal gene transfer, but after analyzing the sequence of the whole region, we realized that the entire contig corresponded to the bacterial genus *Acinetobacter* and that it was therefore more likely to be contamination. In fact, this contig partially aligned with genomes of the species *A. guillouiae* at 97–98% identity. Furthermore, the contig contained a prophage integrated in the *ssrA* gene, which was unknown and, therefore, does not align with any of the 42 bacterial genomes of *A. guillouiae* sequenced to date (Fig. 1a).

Once we had identified the contaminating bacteria, we carried out new similarity searches with all the contigs of the mite assembly against the available genomes of *A. guillouiae*. Thus, we confirmed at least 41 contaminated contigs, spanning a total length of 2.28 Mb (Fig. 1b). This covered 49% of the 4.62 Mb length of the closest genome of *A. guillouiae*.

This error has been propagated on at least one occasion, when, in 2023, another group was investigating the *anmK* gene of *A. baumannii*, anhydro-*N*-acetylmuramic acid kinase, related to antibiotic resistance [28]. They found homologues of this gene in several bacterial genera, and upon finding it in *Oppiella*, they mistook it for a bacterium.

The contaminated contigs contain 491 annotated genes and 20 pseudogenes of *O. nova*, which are artefacts. Therefore, the number of 23,761 mite genes reported by the authors [8] should be reduced to at least 23,250. On the other hand, we propose that the *O. nova* genome should be re-assembled and contaminated contigs should be removed from the genome database.

### *Acinetobacter* is not found in mites from the same family

**Fig. 2.**
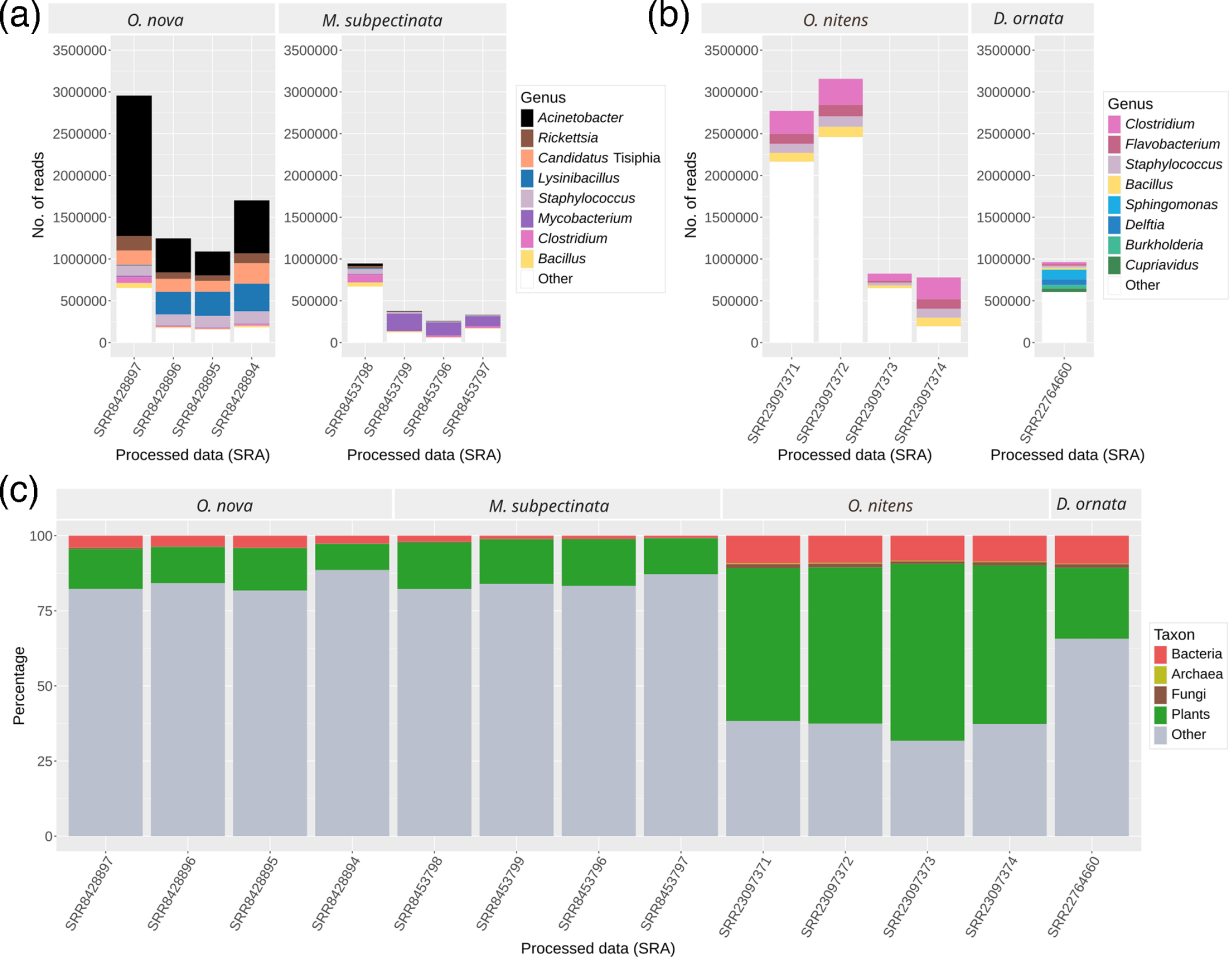
Contamination in mite genomes and assembly of the *A. guillouiae* genome. Number of potential bacterial reads separated by genus from the sequencing of (**a**) *O. nova* and *M. subpectinata* (SRA datasets are sorted by insert length: 180, 350, 550 and 3,000 bp) and (**b**) *Oppia nitens* and *Dissorhina ornata*. Only genera whose reads accounted for more than 0.01% of total reads were considered. (**c**) Proportion of reads classified for the taxa archaea, bacteria, fungi and plants. ‘Other’ includes reads that are not classified in any of the aforementioned taxa.

To track the contamination source, we analysed the raw data from the *O. nova* sequencing. This was carried out by Brandt *et al*. using 4 libraries with different insert sizes (180, 350, 550 and 3,000 bp), the shortest size being used for the initial assembly and the rest to fill gaps and obtain more robust scaffolds [8]. After filtering and analysing the reads, we confirmed the contamination by *Acinetobacter*, as well as a lower level of contamination from the Rickettsiaceae family (genera *Rickettsia* and *Candidatus* Tisiphia) (Fig. 2a). In their article, Brandt *et al*. also sequenced the genome of *M. subpectinata*. In this other species, we found a low level of bacterial reads in the data of the corresponding sequencing.

Currently, sequencing data exist for two other species of mites belonging to the same family, Oppiidae: *O. nitens* [29] and *Dissorhina ornata* [30]. We therefore wanted to determine whether these sequences were contaminated with bacteria. Thus, following the same protocol, evidence of contamination was found, but to a lesser extent, and it did not include *Acinetobacter* or Rickettsiaceae (Fig. 2b).

To further assess the level of contamination of the different sequences, we searched for possible sequences of archaea, fungi and plants, in addition to the corresponding bacteria. In this way, we were also able to confirm additional high contamination of plant sequences, especially in the data of *O. nitens* (Fig. 2c). This contamination caused by the taxon Viridiplantae was unexpected, as the raw data had been cleaned and filtered to remove external contamination. However, the contamination does not appear to be concentrated in specific taxonomic groups, so it could be due to differences in the treatment of *O. nitens* reads and to intrinsic limitations of the mapping method.

### The assembly of two new bacteria

**Fig. 3.**
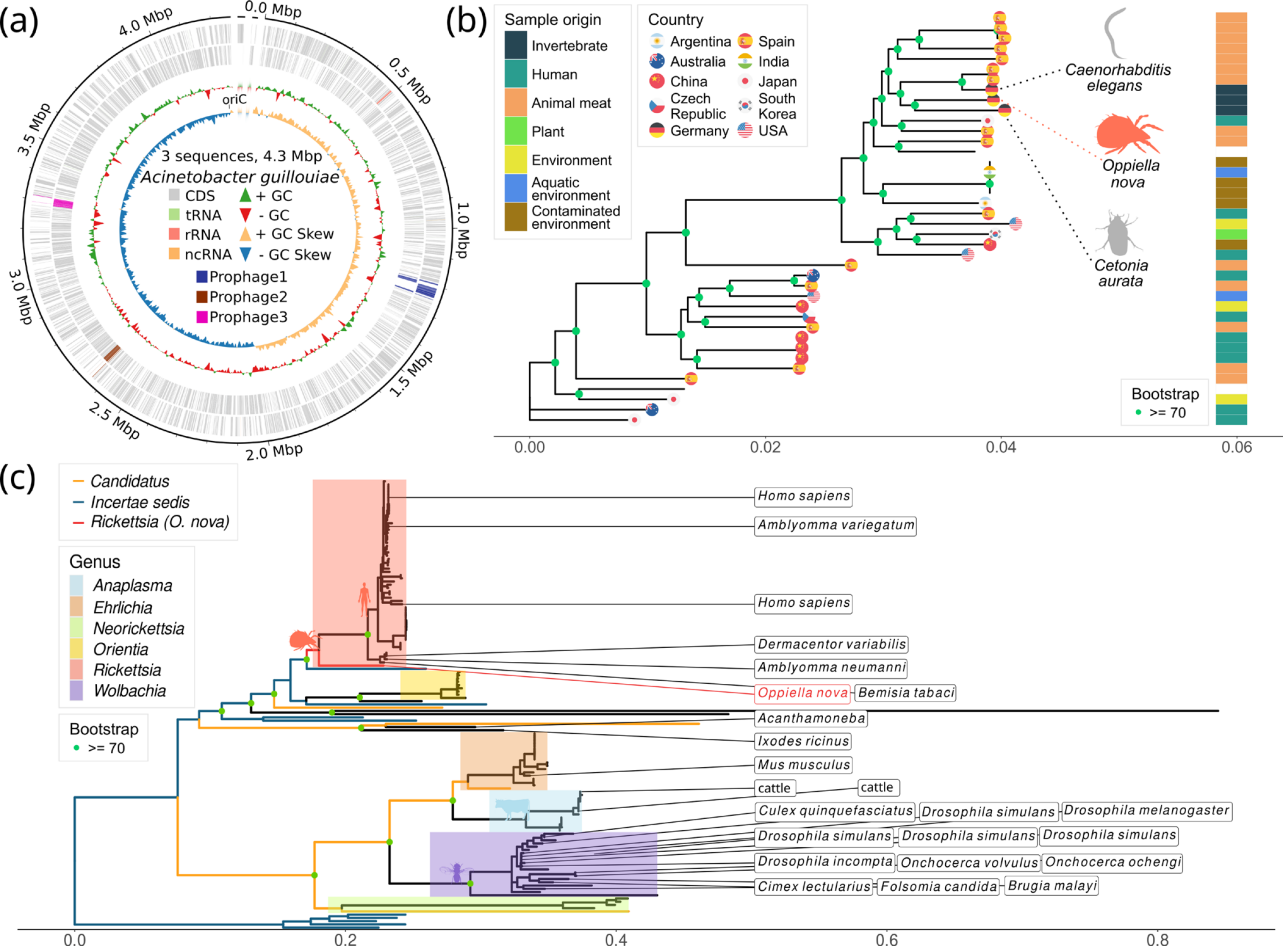
Assembly of the *A. guillouiae* genome and molecular phylogenies of *A. guillouiae* and *Rickettsiales*. (**a**) Assembly of the *A. guillouiae* genome (53× coverage). Three prophages are highlighted: prophage 3 is the same as in Fig. 1(a), and prophage 2 contains a gene encoding a putative glucuronan lyase. (**b**) Phylogenetic tree with the sequenced genomes of *A. guillouiae* including the new genome assembled here. (**c**) Phylogenetic tree of *Rickettsiales* 23S rRNA including the corresponding sequence coming from *O. nova*.

Based on the high level of *Acinetobacter* contamination in *O. nova*, we decided to assemble the genome of *A. guillouiae* from the processed data. We obtained an assembly with 1 long contig of 4.27 Mb, and 2 more of 23 and 17 kb, with an estimated completeness of 95.2% (Fig. 3a). This genome had 4,058 genes and a total of 3 integrated phages (BioSample: SAMN51336641). One of these prophages carries a gene encoding a putative glucuronan lyase (InterPro: IPR025975), which does not appear in any other genome of the species. Given that *Oppiella* is fungivorous, this could suggest a symbiotic relationship between the bacterium and the mite.

Next, we analyzed the evolutionary closeness of this new genome with the rest of the sequenced genomes of the species by means of a molecular phylogeny. The result showed that the new genome of *A. guillouiae* appears close to others associated with invertebrate hosts (Fig. 3b). Finally, with the bacterial reads, we were able to also identify another bacterial species of the genus *Rickettsia*, with an estimated completeness of 80.6% and N50=32,787 bp. The sequence of this new genome is evolutionarily very close to that of two other bacteria of the same genus isolated from ticks (Fig. 3c).

## Discussion

Here, we report the case of the assembly of a genome of the mite *O. nova*, in which bacterial contamination went unnoticed, which has led to the error beginning to spread in the scientific literature [28]. This error occurred due to the presence of bacteria of at least *A. guillouiae* in the mite samples, and it could have gone undetected due to contamination in the public metazoan database used to filter the contigs or for some other unknown reason.

Bacteria of the genus *Acinetobacter* have previously been associated with various species of arthropods. For instance, *A. baumannii* has been isolated from the intestine of the planthopper *Nilaparvata lugens* and has been shown to provide protection against a pathogenic fungus [31]. In addition, *Acinetobacter* has emerged as the dominant genus in flocculent fibrous dust associated with mites of the genus *Dermatophagoides* [32]. Remarkably, when these household mites are fed with dead bodies of mites, faeces and diet debris, the amount of *Acinetobacter* in their guts increases [33]. Finally, in a mite that feeds on the blood of poultry, *Dermanyssus gallinae*, it has been observed that when it is subjected to starvation, *Acinetobacter* becomes dominant, among other genera, in its intestinal microbiota [34].

Here, we have found evidence of a microbiota associated with the mite *O. nova*, dominated by *A. guillouiae*. Since the mites were starved for at least a week before their genomes were extracted [8], this could be the cause of the increased bacterial contamination found in the processed data, as described in the case of *D. gallinae*.

It should also be considered that the orphan glucuronan lyase found in a bacteriophage integrated into the genome assembled here would point to a phage-bacterium-mite symbiosis. This enzyme could be involved in the targeting and cleavage of linear β-(1,4)-polyglucuronic acid from fungi or other bacteria, with the aim of breaking it down into smaller sugars and using it as an energy source [35]. However, this enzyme has also been found in other symbiotic and pathogenic bacteria, which may use it to degrade glucuronan present in the extracellular matrix or cell walls of their eukaryotic hosts or other microbes, facilitating colonization, invasion or nutrient uptake [36].

In *O. nova*, only the presence of endosymbiotic bacteria of the genera *Wolbachia* and *Cardinium*, common in arthropods, had been previously observed [37,38]. However, the authors emphasized that many questions remain unanswered regarding the role of bacterial symbiosis in this mite. Something that supports this association is the fact that *A. guillouiae* has been linked to oil-contaminated soil [39], as is the case with *O. nova* [40].

On the other hand, the *Rickettsia* found in the *O. nova* assembly could also be an endosymbiont of the mite. In book lice, *Rickettsia* is present in all asexually reproducing individuals, but not in sexually reproducing strains [41]. Thus, the fact that this bacterium does not appear in the closely related *M. subpectinata*, which has sexual reproduction, allows us to propose that the *Rickettsia* species could be causing *O. nova* to reproduce asexually.

For the rest of the mite species analysed, no conclusive results on bacterial contamination could be obtained. Although sequences from other bacterial species such as *Mycobacterium* appear in some of them, a finding also reported for the chigger mite *Leptotrombidium imphalum* [42].

In conclusion, here, we show that data from whole-genome sequencing projects stored in public databases could enable the future discovery of new bacteria in the microbiome of arthropods.

## Supplementary material

10.1099/mgen.0.001520 Uncited Supplementary Material 1.

## References

[1] Boothby TC, Tenlen JR, Smith FW, Wang JR, Patanella KA (2015). Evidence for extensive horizontal gene transfer from the draft genome of a tardigrade. Proc Natl Acad Sci USA.

[2] Arakawa K (2016). No evidence for extensive horizontal gene transfer from the draft genome of a tardigrade. Proc Natl Acad Sci USA.

[3] Bemm F, Weiß CL, Schultz J, Förster F (2016). Genome of a tardigrade: horizontal gene transfer or bacterial contamination?. Proc Natl Acad Sci USA.

[4] Rubio A, Mier P, Andrade-Navarro MA, Garzón A, Jiménez J (2020). CRISPR sequences are sometimes erroneously translated and can contaminate public databases with spurious proteins containing spaced repeats. Database.

[5] Lu J, Salzberg SL (2018). Removing contaminants from databases of draft genomes. PLoS Comput Biol.

[6] Francois CM, Durand F, Figuet E, Galtier N (2020). Prevalence and implications of contamination in public genomic resources: a case study of 43 reference arthropod assemblies. G3.

[7] Rubio A, Garzón A, Moreno-Rodríguez A, Pérez-Pulido AJ (2024). Biological warfare between two bacterial viruses in a defense archipelago sheds light on the spread of CRISPR-Cas systems. Cell Rep.

[8] Brandt A, Tran Van P, Bluhm C, Anselmetti Y, Dumas Z (2021). Haplotype divergence supports long-term asexuality in the oribatid mite *Oppiella nova*. Proc Natl Acad Sci USA.

[9] Ponge JF (1991). Food resources and diets of soil animals in a small area of Scots pine litter. Geoderma.

[10] Schwengers O, Jelonek L, Dieckmann MA, Beyvers S, Blom J (2021). Bakta: rapid and standardized annotation of bacterial genomes via alignment-free sequence identification. Microb Genom.

[11] Wishart DS, Han S, Saha S, Oler E, Peters H (2023). PHASTEST: faster than PHASTER, better than PHAST. Nucleic Acids Res.

[12] Marçais G, Delcher AL, Phillippy AM, Coston R, Salzberg SL (2018). MUMmer4: a fast and versatile genome alignment system. PLoS Comput Biol.

[13] Bolger AM, Lohse M, Usadel B (2014). Trimmomatic: a flexible trimmer for Illumina sequence data. Bioinformatics.

[14] Chen S, Zhou Y, Chen Y, Gu J (2018). fastp: an ultra-fast all-in-one FASTQ preprocessor. Bioinformatics.

[15] O’Connell J, Schulz-Trieglaff O, Carlson E, Hims MM, Gormley NA (2015). NxTrim: optimized trimming of Illumina mate pair reads. Bioinformatics.

[16] Wood DE, Lu J, Langmead B (2019). Improved metagenomic analysis with Kraken 2. Genome Biol.

[17] Kim D, Langmead B, Salzberg SL (2015). HISAT: a fast spliced aligner with low memory requirements. Nat Methods.

[18] Li H, Handsaker B, Wysoker A, Fennell T, Ruan J (2009). The sequence alignment/map format and SAMtools. Bioinformatics.

[19] Wick RR, Judd LM, Gorrie CL, Holt KE (2017). Unicycler: resolving bacterial genome assemblies from short and long sequencing reads. PLoS Comput Biol.

[20] Cantalapiedra CP, Hernández-Plaza A, Letunic I, Bork P, Huerta-Cepas J (2021). eggNOG-mapper v2: functional annotation, orthology assignments, and domain prediction at the metagenomic scale. Mol Biol Evol.

[21] Alonge M, Lebeigle L, Kirsche M, Jenike K, Ou S (2022). Automated assembly scaffolding using RagTag elevates a new tomato system for high-throughput genome editing. Genome Biol.

[22] Manni M, Berkeley MR, Seppey M, Simão FA, Zdobnov EM (2021). BUSCO update: novel and streamlined workflows along with broader and deeper phylogenetic coverage for scoring of eukaryotic, prokaryotic, and viral genomes. Mol Biol Evol.

[23] Tonkin-Hill G, MacAlasdair N, Ruis C, Weimann A, Horesh G (2020). Producing polished prokaryotic pangenomes with the Panaroo pipeline. Genome Biol.

[24] Katoh K, Standley DM (2013). MAFFT multiple sequence alignment software version 7: improvements in performance and usability. Mol Biol Evol.

[25] Minh BQ, Schmidt HA, Chernomor O, Schrempf D, Woodhams MD (2020). IQ-TREE 2: new models and efficient methods for phylogenetic inference in the genomic era. Mol Biol Evol.

[26] Quast C, Pruesse E, Yilmaz P, Gerken J, Schweer T (2013). The SILVA ribosomal RNA gene database project: improved data processing and web-based tools. Nucleic Acids Res.

[27] Towner K, Dworkin M, Falkow S, Rosenberg E, Schleifer KH, Stackebrandt E (2006). The Prokaryotes: A Handbook on the Biology of Bacteria Volume 6: Proteobacteria: Gamma Subclass.

[28] Gupta D, Mondal P (2023). Drug repurposing against anhydro-N-acetylmuramic acid kinase of multi-drug resistant *Acinetobacter baumannii*: an *in silico* approach. Biosci Biotech Res Asia.

[29] Adedokun AA, Fajana HO, Jegede OO, Hammond AS, Smith DDN (2024). Exploring the genome of the oribatid mite, *Oppia nitens*—environmental stress response and toxicity adaptation. biorxiv.

[30] Collins G, Schneider C, Boštjančić LL, Burkhardt U, Christian A (2023). The MetaInvert soil invertebrate genome resource provides insights into below-ground biodiversity and evolution. Commun Biol.

[31] Tang C, Hu X, Tang J, Wang L, Liu X (2024). The symbiont *Acinetobacter baumannii* enhances the insect host resistance to entomopathogenic fungus *Metarhizium anisopliae*. *Commun Biol*.

[32] Li Z, Zheng N, An Q, Li X, Sun S (2022). Impact of environmental factors and bacterial interactions on dust mite allergens in different indoor dust. Sci Total Environ.

[33] Molva V, Bostlova M, Nesvorna M, Hubert J (2020). Do the microorganisms from laboratory culture spent growth medium affect house dust mite fitness and microbiome composition?. Insect Sci.

[34] Liu Q, Sun T, Wang P, Wang L, Frantova H (2024). Significant role of symbiotic bacteria in the blood digestion and reproduction of *Dermanyssus gallinae* mites. ISME Commun.

[35] Pilgaard B, Vuillemin M, Munk L, Holck J, Meier S (2022). Discovery of a novel glucuronan lyase system in *Trichoderma parareesei*. Appl Environ Microbiol.

[36] MacDonald LC, Berger BW (2014). A polysaccharide lyase from *Stenotrophomonas maltophilia* with a unique, pH-regulated substrate specificity. J Biol Chem.

[37] Konecka E, Olszanowski Z (2015). A screen of maternally inherited microbial endosymbionts in oribatid mites (*Acari: Oribatida*). Microbiology.

[38] Konecka E (2022). Fifty shades of bacterial endosymbionts and some of them still remain a mystery: *Wolbachia* and *Cardinium* in oribatid mites (*Acari: Oribatida*). J Invertebr Pathol.

[39] Kim HR, Lee C, Shin H, Kim J, Jeong M (2023). Isolation of a polyethylene-degrading bacterium, *Acinetobacter guillouiae*, using a novel screening method based on a redox indicator. Heliyon.

[40] Melekhina EN, Belykh ES, Markarova MY, Taskaeva AA, Rasova EE (2021). Soil microbiota and microarthropod communities in oil contaminated sites in the European Subarctic. Sci Rep.

[41] Yang Q, Kučerová Z, Perlman SJ, Opit GP, Mockford EL (2015). Morphological and molecular characterization of a sexually reproducing colony of the booklouse Liposcelis bostrychophila (*Psocodea: Liposcelididae*) found in Arizona. Sci Rep.

[42] Ponnusamy L, Willcox AC, Roe RM, Davidson SA, Linsuwanon P (2018). Bacterial microbiome of the chigger mite *Leptotrombidium imphalum* varies by life stage and infection with the scrub typhus pathogen *Orientia tsutsugamushi*. PLoS One.

