# Supplementary material for: Discovery of a bacteriophage sequence in a mite genome assembly reveals bacterial contamination and opens new possibilities for exploring arthropod symbionts
Source: Microb Genom. 2025 Oct 8;11(10):001520. doi: 10.1099/mgen.0.001520 (PMC12507336; doi:10.1099/mgen.0.001520)
Supplement: Uncited Supplementary Material 1. [file mgen-11-01520-s001.pdf]

A bacteriophage in a mite genome reveals bacterial contamination and opens new possibilities for exploring arthropod symbionts

Supplementary Material

Elisa Luque-Jiménez[1], Antonio Moreno-Rodríguez[1], Andrés Garzón[1], Alejandro Rubio[1], Antonio J. Pérez-Pulido[1]

[1]Andalusian Centre for Developmental Biology (CABD, UPO-CSIC-JA). Faculty of Experimental Sciences (Genetics Area), University Pablo de Olavide, 41013, Seville, Spain

Supplementary Materials and Methods

Search for contaminated contigs

To find contaminated contigs, the sequences of the *Oppiella nova* assembly were compared with the 42 available *Acinetobacter guillouiae* assemblies. A contig of a minimal length of 3.5 kb was considered to belong to *A. guillouiae* when 90% of its sequence showed at least 95% similarity to a sequence of the bacterium.

To validate these contigs and to visualize the results, all the *O. nova* contigs were compared against *A. guillouiae* strain 13-3 (GenBank: CP142740.1), using MUMmer v4.0.1 (1):

- `nucmer CP142740.fna GCA_905397405.1_1_On_b1v03.max_arth_b2g_droso_b2g_emblv2_genomic.fna`

To visualize the results, a modified version of the R script MUMmerplots with ggplot2 was used, available at:

- https://jmonlong.github.io/Hippocamplus/2017/09/19/mummerplots-with-ggplot2/

Analysis of the raw data

To identify the origin of the contamination, we first downloaded raw data from the original manuscripts for *Oppiella nova* and *Medioppia subpectinata* (2), *Oppia nitens* (3) and *Dissorhina ornata* (4), which were retrieved using the NCBI fasterq-dump tool v3.2.0. Then we used Kraken v2.1.2, a tool that associates sequences with taxa of a given database (5). It is designed to work with either paired end or single end reads. The DNA libraries containing the raw data were made up of unfiltered paired end reads except for two, which contained mate paired reads (insert size 3 kb). In order to use Kraken2 with the raw data and the mate paired reads, we had to filter and process them.

The mate paired data were processed according with Illumina guidelines using the Nxtrim tool v0.4.3 (6), which is designed to remove adapters from this specific type of sequencing and to categorize reads according to their orientation:

- `fasterq-dump --split-files SRR8428894`
- `nxtrim -1 SRR8428894_1.fastq -2 SRR8428894_2.fastq -O SRR8428894_all_trim_nx --separate --preserve-mp --minlength 40`

After creating these groups, poor-quality reads, in all raw datasets, were eliminated according with the parameters established by the authors of the original manuscripts using either the trimmomatic tool v0.39 (7) for the *Oppiella nova*, *Medioppia subpectinata* and *Dissorhina ornata* reads (parameters for adapter removal have not been included), or the fastp tool v1.0.1 (8) for the *Oppia nitens* reads:

- `trimmomatic PE -threads 20 SRR8428897_1.fastq SRR8428897_2.fastq trim1_ SRR8428897.fastq untrim1_ SRR8428897.fastq trim2 SRR8428897.fastq untrim2_ SRR8428897.fastq LEADING:20 TRAILING:20 SLIDINGWINDOW:3:20 MINLEN:100`

- `trimmomatic PE -threads 20 SRR8428894_all_trim_nx_R1.pe.fastq.gz SRR8428894_all_trim_nx_R2.pe.fastq.gz trim1_pe.fastq untrim1_pe.fastq trim2_pe.fastq untrim2_pe.fastq LEADING:20 TRAILING:20 SLIDINGWINDOW:4:20 MINLEN:60`

- `fastp -i SRR23097371_1.fastq.gz -I SRR23097371_2.fastq.gz -o SRR23097371_1_fastp.fastq.gz -O SRR23097371_2_fastp.fastq.gz -w 20`

Following the processing of the filtered data, we used Kraken2 for the identification of bacterial taxa. We initially used a pre-built Kraken2 specific database which contained all RefSeq annotated bacteria sequences from the NCBI Genome database. Then, we obtained the putative taxa of all the paired end reads of the filtered data.

- `kraken2-build --download-taxonomy --db db_bacterias`
- `kraken2-build --download-library bacteria --db db_bacterias`
- `kraken2-build --build --db db_bacterias`
- `kraken2 --db db_bacterias --output SRR8428894_pe_output.txt --report SRR8428894_pe_report.txt --classified-out SRR8428894_pe_classified#.txt --use-names --paired SRR8428894_pe_1.fastq SRR8428894_pe_2.fastq`

Additionally, we used Kraken2 for the identification of plant, fungi and archaea taxa, using the pre-built RefSeq annotated sequences database libraries available from this software. We executed the same commands lines as with the bacterial database.

Assembly of the bacterial genome

Initially, all contigs that were deemed to be contaminated by *A. guillouiae* were removed from the *O. nova* reference genome (refer to the section "Search for contaminated contigs"). The filtered genome was then indexed using the HISAT2 tool v2.2.1 (9):

- `hisat2-build onova_filter.fasta onova_only`

Filtered reads were mapped against the *O. nova* genome, excluding contigs contaminated by *A. guillouiae*. The HISAT2 algorithm v2.2.1 was used for this purpose. Later, reads that were not mapped were subjected to positive selection with the SAMtools tool v1.21 (10). This process resulted in the discarding of all reads belonging to *O. nova* and other taxa different to *Acinetobacter*:

- `hisat2 -q -p 96 -x onova_only -1 trim1.fastq -2 trim2.fastq --no-spliced-alignment -S SRR8428897_onova_only.sam`
- `samtools view --threads 96 -Sb -f 4 SRR8428897_onova_only.sam > SRR8428897_notonova.bam`

Before assembly, the bam file was converted to fastq format using BEDTools v2.31.1 (11). Next, a *de novo* assembly was executed employing the Unicycler algorithm v0.5.1 (12), with the default parameters, to ensure optimal performance:

- `bamToFastq -i SRR8428897_notonova.bam -fq reads_not_onova_1.fastq -fq2 reads_not_onova_2.fastq`
- `unicycler -t 96 -1 reads_not_onova_1.fastq -2 reads_not_onova_2.fastq -o unicycler --verbosity 1 --min_fasta_length 100 --mode normal`

Once a draft genome was obtained, gene prediction was performed using Bakta v1.10.3 (13). These predicted genes were then functionally annotated with eggNOG-mapper v2.1.12 (14), due to the suspected mixing of genomes from different bacterial species. This functional annotation was then used to filter the initial contigs, employing a PERL language script, into three distinct groups: *A. guillouiae*, Rickettsiales, and others:

- `bakta --db db_bakta/db-full/ --output bakta_Aguillo --genus Acinetobacter --prefix Agui --locus-tag Agui --threads 96 unicycler/assembly.fasta`
- `emapper.py -i bakta_Aguillo/agui.faa -o annot --cpu 20 –override`

- `perl filter_annot.pl annot.emapper.annotations agui.gff3 agui.fna`

To achieve a complete genome, *Moraxellaceae* family-specific contigs were used in comparison with closed genomes of *A. guillouiae*. This scaffolding was performed with the RagTag tool v2.1.0 (15). A new gene prediction and subsequent functional annotation of genes was performed on the final genome according to the previously established methodology:

- `ragtag.py scaffold ../unicycler_filter/GCA_020172725.1_ASM2017272v1_genomic.fna genomics_agui_1kb.fna`
- `bakta --db db_bakta/db-full/ --output bakta_Aguillo_def --genus Acinetobacter --prefix Agui --locus-tag Agui --threads 96 ragtag_output/ragtag.scaffold.fasta`
- `emapper.py -i bakta_Aguillo_def/agui.faa -o annot_def --cpu 20 --override`

Molecular phylogenies

For the molecular phylogeny of *A. guillouiae*, three genomes labeled with "genome length too small" in the NCBI Genome database were discarded. The remaining 39 genomes were annotated using Bakta as previously described.

- `bakta -d bakta_db -o bakta_output/ GCF_000829655.1_ASM82965v1_genomic -f GCF_000829655.1_ASM82965v1_genomic.fna --prefix GCF_000829655.1_ASM82965v1_genomic --genus Acinetobacter --locus-tag 000829655`

We create the bacterial pangenome of *A. guillouiae* to select core genes. For that, we took the annotated genomes from Bakta output and used Panaroo v1.5.2 to generate a core genes alignment (16), with MAFFT as the designated aligner.

- `panaroo -i bakta/output/* -o panaroo --clean-mode strict -a core --aligner mafft`

A phylogeny was then built using the IQ-TREE algorithm v2.4.0 with bootstrap 1000 (17), based on the aligned core genome obtained from Panaroo.

- `iqtree -s panaroo/core_gene_alignment_filtered_cut_id.phy -B 1000 -alrt 1000 -redo`

To build the phylogeny of the order Rickettsiales, firstly 23S ribosomal RNA sequences with any annotation related to mitochondria were discarded, and all those belonging to this taxon were searched in the SILVA v.138.2_LSURef_NR99 database (18).

All these 23S rRNA were compared against contigs classified as Rickettsiales from *O. nova* by using BLASTn v.2.16.0. The best hit was identified as the sequence of 23S rRNA of this Rickettsia.

- `makeblastdb -in contigs_ricket.fna -dbtype nucl`
- `blastn –query SILVA_138.2_LSURef_NR99_tax_silva.fasta -db contigs_ricket.fna -outfmt '6 qseqid sseqid pident qcovs qcovhsp length qlen slen evalue qstart qend sstart send qseq sseq' -max_target_seqs 1 -num_threads 10`

Using this sequence and the rest of the 23S rRNA sequences of the taxon Rickettsiales, a multiple alignment was made using MAFFT v.7.526 (19). The phylogenetic tree was constructed from this alignment by IQ-TREE, using a bootstrap of 1000 and the model *GTR+F+I+G4*.

- `/usr/bin/mafft  --globalpair --maxiterate 16 --phylipout --reorder 23S_Ricke.fasta > 23S_Ricke.mafft`
- `iqtree -s 23S_Ricke.mafft -st DNA -m GTR+F+I+G4 -bb 1000 -wbt -alrt 1000`

To create figures 3B and 3C, IQ-TREE output was drawn using R packages ggtree v3.12.0 and ggimage v0.3.3.

References

1. G. Marçais, *et al.*, MUMmer4: A fast and versatile genome alignment system. *PLOS Computational Biology* **14**, e1005944 (2018).

2. A. Brandt, *et al.*, Haplotype divergence supports long-term asexuality in the oribatid mite Oppiella nova. *Proc Natl Acad Sci U S A* **118**, e2101485118 (2021).

3. A. A. Adedokun, *et al.*, Exploring The Genome of The Oribatid Mite, Oppia Nitens – Environmental Stress Response and Toxicity Adaptation. [Preprint] (2024). Available at: https://www.biorxiv.org/content/10.1101/2024.10.27.619232v1 [Accessed 16 July 2025].

4. G. Collins, *et al.*, The MetaInvert soil invertebrate genome resource provides insights into below-ground biodiversity and evolution. *Commun Biol* **6**, 1241 (2023).

5. D. E. Wood, J. Lu, B. Langmead, Improved metagenomic analysis with Kraken 2. *Genome Biology* **20**, 257 (2019).

6. J. O’Connell, *et al.*, NxTrim: optimized trimming of Illumina mate pair reads. *Bioinformatics* **31**, 2035–2037 (2015).

7. A. M. Bolger, M. Lohse, B. Usadel, Trimmomatic: a flexible trimmer for Illumina sequence data. *Bioinformatics* **30**, 2114–2120 (2014).

8. S. Chen, Y. Zhou, Y. Chen, J. Gu, fastp: an ultra-fast all-in-one FASTQ preprocessor. *Bioinformatics* **34**, i884–i890 (2018).

9. D. Kim, B. Langmead, S. L. Salzberg, HISAT: a fast spliced aligner with low memory requirements. *Nat Methods* **12**, 357–360 (2015).

10. H. Li, *et al.*, The Sequence Alignment/Map format and SAMtools. *Bioinformatics* **25**, 2078–2079 (2009).

11. A. R. Quinlan, I. M. Hall, BEDTools: a flexible suite of utilities for comparing genomic features. *Bioinformatics* **26**, 841–842 (2010).

12. R. R. Wick, L. M. Judd, C. L. Gorrie, K. E. Holt, Unicycler: Resolving bacterial genome assemblies from short and long sequencing reads. *PLOS Computational Biology* **13**, e1005595 (2017).

13. O. Schwengers, *et al.*, Bakta: rapid and standardized annotation of bacterial genomes via alignment-free sequence identification. *Microbial Genomics* **7**, 000685 (2021).

14. C. P. Cantalapiedra, A. Hernández-Plaza, I. Letunic, P. Bork, J. Huerta-Cepas, eggNOG-mapper v2: Functional Annotation, Orthology Assignments, and Domain Prediction at the Metagenomic Scale. *Molecular Biology and Evolution* **38**, 5825–5829 (2021).

15. M. Alonge, *et al.*, Automated assembly scaffolding using RagTag elevates a new tomato system for high-throughput genome editing. *Genome Biol* **23**, 258 (2022).

16. G. Tonkin-Hill, *et al.*, Producing polished prokaryotic pangenomes with the Panaroo pipeline. *Genome Biology* **21**, 180 (2020).

17. B. Q. Minh, *et al.*, IQ-TREE 2: New Models and Efficient Methods for Phylogenetic Inference in the Genomic Era. *Mol Biol Evol* **37**, 1530–1534 (2020).

18. C. Quast, *et al.*, The SILVA ribosomal RNA gene database project: improved data processing and web-based tools. *Nucleic Acids Res* **41**, D590-596 (2013).

19. K. Katoh, D. M. Standley, MAFFT multiple sequence alignment software version 7: improvements in performance and usability. *Mol. Biol. Evol.* **30**, 772–780 (2013).
